# TIGIT and SNRPA1 as novel diagnostic and predictive biomarkers in obstructive ventilatory dysfunction combined with pulmonary nontuberculous mycobacterial infection patients

**DOI:** 10.3389/fcimb.2025.1621129

**Published:** 2025-10-01

**Authors:** Minlong Zhang, Cuiping Yang, Yinghua Guo

**Affiliations:** College of Pulmonary & Critical Care Medicine, 8^th^Medical Centre, Chinese People's Liberation Army (PLA) General Hospital, Beijing, China

**Keywords:** biomarker, NTM, obstructive ventilatory dysfunction, machine learning, diagnostic model

## Abstract

**Background:**

Patients with obstructive ventilatory dysfunction are prone to NTM (nontuberculous mycobacterial) colonization and infection. Our investigation employs an integrative bioinformatics approach to elucidate critical molecular signatures linked to obstructive ventilatory dysfunction combined with NTM infection, and constructing a clinical diagnostic model using core differentially expressed genes.

**Methods:**

The GSE97298 dataset from the GEO database was analyzed via GEO2R to identify differentially expressed genes (DEGs). Enrichment analysis of the DEGs was conducted using Gene Ontology (GO), Kyoto Encyclopedia of Genes and Genomes (KEGG) enrichment analyses and Gene Set Enrichment Analysis (GSEA). DEGs were further conducted immune infiltration analysis and screened for core genes through protein–protein interaction (PPI) network analysis and machine learning (Lasso regression and Random Forest). The DSigDB database was employed to explore the potential targeted drugs of characteristic genes. Diagnostic potential and predictive model of candidate biomarkers was assessed through five machine learning (ML) techniques, namely XGBoost, LightGBM, Random Forest (RF), Adaptive Boosting (Adaboost), and Support Vector Machine (SVM) modeling. Shapley Additive Interpretation (SHAP) analysis facilitated a visual interpretation for individual patient.

**Results:**

A total of 69 DEGs, which are widely involved in important biological processes such as cell cycle, tubulin binding, and RNA processing, were identified. Immune cell analysis indicated that B cells, T follicular helper cells and T Cells were positively correlated with NTM, Mast cells, Macrophages cells and NK CD56dim cells showed a negative correlation. Through PPI network analysis and machine learning, TIGIT and SNRPA1 were selected as the core gene and significant predictors for subsequent analysis. The expression of TIGIT and SNRPA1 proteins in NTM patients’ blood samples were also down-regulated compared with control. Using the DSigDB database, we predicted seven drugs that exhibit significant binding activity with core genes. Importantly, SNRPA1/TIGIT was the optimal combination for predicting obstructive ventilatory dysfunction combined with NTM infection. The RF model surpassing the performance of other models. SHAP analysis provided independent explanations, reaffirming the critical factors associated with the risk of obstructive ventilatory dysfunction combined with NTM infection.

**Conclusion:**

Our study demonstrated that TIGIT and SNRPA1 were down-regulated and were strongly associated with NTM infection. In addition, we successfully established a precise predictive model for risk of obstructive ventilatory dysfunction combined with NTM infection using machine learning techniques, offering valuable support to clinicians in making informed clinical decisions.

## Introduction

NTM (nontuberculous mycobacteria) is a broad group of environmental organisms that can transiently colonize human airways without necessarily causing disease. Pulmonary NTM (pNTM) occurs when these organisms establish symptomatic, progressive infection—typically in lungs with structural or functional impairment—leading to radiologic changes and clinical illness. Thus, NTM exposure/colonization is the prerequisite; pNTM represents the pathogenic consequence in susceptible hosts. pNTM disease is an increasingly common and challenging infection. Although these mycobacteria are ubiquitous in the environment, clinical disease typically occurs in individuals with preexisting pulmonary conditions such as bronchiectasis or obstructive ventilatory dysfunction (Choi et al., 2024; Dhasmana et al., 2024; Lapinel et al., 2024; Baig et al., 2025). However, a subgroup of patients develop pNTM disease without any known risk factors. The condition is associated with significant mortality, ranging from 12.5% to 41.1% within 5 years (André et al., 2024; Kim et al., 2024; Lee et al., 2024). The clinical course of pNTM disease is highly variable and often unpredictable; while some patients experience progressive disease, others may remain stable without treatment. The factors influencing disease acquisition and its subsequent progression remain poorly understood.

Obstructive ventilatory dysfunction (OVD) patients face a disproportionate burden of NTM infection, with prevalence exceeding 12% and mortality doubled. Established risk factors include bronchiectasis extent, emphysema severity, and corticosteroid load, yet existing prediction tools perform poorly. No transcriptomic model specific to OVD exists. Our study fills this gap by integrating clinical and multi-omics data to deliver the first validated, biologically-informed NTM risk predictor for OVD patients.

Disseminated NTM disease is often associated with immunodeficiency, such as in acquired immunodeficiency syndrome or in individuals with inherited or acquired defects in the IFN-γ/IL-12 pathway. Additionally, several studies have identified functional immune defects in patients with pNTM disease (Hashemzadeh et al., 2025; Liang et al., 2025). A recent study found a higher prevalence of genetic variants affecting immune response-related genes in pNTM patients compared to controls and unaffected family members (Cowman et al., 2018; Prieto et al., 2022). These results indicate that impaired immune responses may contribute to the susceptibility to pNTM disease. In addition, clinical and radiological features of pNTM disease often overlap with those of pulmonary tuberculosis (PTB) and bacterial pneumonia, resulting in misdiagnosis or delayed treatment. To overcome the long turnaround time and limited sensitivity of traditional culture- and molecular-based assays, researchers integrated serum Olink proteomics with lipidomics to map immune-metabolic differences between NTM and MTB infections, identify joint biomarkers, and build an accurate multi-omics diagnostic model (Wang et al., 2024). These results underscore the critical importance of multi-omics approaches and machine learning in NTM infection research.

In this study, we applied multiple bioinformatics analyses and ultimately identified two core genes. We predicted seven drugs using molecular docking method. We then leveraged these markers to build diagnostic models with multiple machine-learning algorithms, improving early detection of patients at high risk for both obstructive ventilatory dysfunction and NTM infection.

## Materials and methods

### Data collection and data processing

The Gene Expression Omnibus database (https://www.ncbi.nlm.nih.gov/geo) were used to obtain clinical data and gene expression profiles. The study design is presented in the form of a flow diagram (**Supplementary Figure S1**). The raw data of GSE97298 (Affymetrix Human Gene 1.1 ST Array; Affymetrix, Santa Clara, CA), with a total of 89 whole blood samples (Based on the clinical information from the dataset, we selected 13 cases with obstructive ventilatory impairment (FEV1/FVC ratio less than 0.7) as controls, and at the same time, chose 12 cases with obstructive ventilatory impairment complicated by NTM infection as the experimental group) were downloaded from the GEO database to screen DEGs. We utilized the platform from GPL11532 to convert the probe-level expression data into gene-level expression data. Probes that corresponded to multiple genes were excluded. When multiple probes were associated with a single gene, their expression values were averaged for subsequent analysis. The microarray data’s probes were then mapped to gene symbols using the Bioconductor Annotation Data package.

### Identification of differentially expressed genes

To pinpoint genes that show significant expression differences, we employed the GEO2R online tool for DEG screening to analyze the dataset (Ritchie et al., 2015). The genes were considered differentially expressed (DEGs) if they met the following criteria: a log2-fold change (FC) exceeding 0.5 or falling below −0.5, coupled with an p-value of less than 0.05. For a clearer representation of these DEGs, we utilized the ggplot2 package to create volcano plots and the pheatmap package to produce heatmaps.

### Enrichment analysis

To explore the biological roles and associated pathways of the DEGs, we performed a comprehensive functional enrichment analysis (Yu et al., 2012). This involved utilizing the clusterProfiler package within R software to carry out Gene Ontology (GO) analysis, which covered aspects such as cellular component, biological process, and molecular function, as well as Kyoto Encyclopedia of Genes and Genomes (KEGG) pathway analysis. We focused on GO terms and pathways that were significantly enriched, defined by an adjusted p-value threshold of less than 0.05. To present these findings in a visually engaging manner, we employed the R packages “enrichplot” and “ggplot2” for data visualization. GO enrichment mainly includes biological processes (BP), molecular functions (MF), and cellular components (CC).

The Gene Set Enrichment Analysis (GSEA) method assesses how genes within a specific set are distributed across a ranked list of genes associated with a particular phenotype. This approach helps to determine the influence of these genes on the phenotype of interest. For our analysis, expression data derived from the “limma” package were employed. We performed GSEA using the “Hallmark gene sets” and the GSEA functionality provided by the “clusterProfiler” R package. This process allowed us to identify significant gene sets that are enriched in our dataset, potentially shedding light on the underlying biological mechanisms related to the phenotype (absolute normalized enrichment score (|NES|) ≥ 1).

### Protein–protein interaction network and gene module identification

We performed a Protein-Protein Interaction (PPI) analysis on the differentially expressed genes (DEGs) using the STRING database (https://cn.string-db.org/), a resource that facilitates the exploration and analysis of both known and predicted protein interactions (Liu et al., 2018). The DEGs were submitted to STRING with specified parameters: network type was set to the comprehensive STRING network, network edges represented confidence levels, and the minimum interaction score was set to the highest confidence threshold of 0.900.

### Selection of core genes via machine learning

The candidate core genes were subjected to further refinement using two distinct machine learning approaches: the Least Absolute Shrinkage and Selection Operator (LASSO) and the Random Forest algorithm. LASSO, a popular technique for feature selection, and Random Forest, which leverages an ensemble of decision trees for classification or regression predictions, were employed for this purpose.

For the LASSO analysis, we utilized the “glmnet” package, configuring it with a binomial response type and an alpha parameter set to 1. To enhance model accuracy, a 10-fold cross-validation strategy was implemented. The lambda.1se (lambda one standard error) parameter, known for its effectiveness in regularization optimization, was selected to prune features. In the case of Random Forest, the “RandomForest” package was used to construct the model, and genes were filtered based on their importance score, with those exceeding a threshold of 1 being selected.

The final list of core genes was determined by intersecting the gene lists obtained from both LASSO and Random Forest screenings, ensuring that only those genes identified as significant by both methods were considered as core genes. This dual-method approach provided a more robust and reliable identification of key genes potentially driving the observed phenotypes.

### Infiltration analysis of immune cells

Single-sample GSEA (ssGSEA) is relying on the improvement of GSEA method, and the gene expression data of each sample is normalized, and then the ssGSEA score corresponding to each gene set is calculated. Specifically, we performed ssGSEA by R packages “GSVA” and “GSEABase” to assess the extent of infiltration of 24 distinct immune cell types (aDC, B cells, CD8 T cells, Cytotoxic cells, DC Eosinophils, iDC, Macrophages, Mast cells, Neutrophils, NK CD56bright cells, NK CD56dim cells, NK cells, pDC, T cells, T helper cells, Tcm, Tem, TFH, Tgd, Th1 cells, Th17 cells, Th2 cells, TReg) within the GSE97298 dataset (the final 25 included samples).

### PCR validation

We recruited three obstructive ventilatory dysfunction (OVD) patients co-infected with NTM and three matched OVD controls from our center. Peripheral blood was drawn into PAXgene tubes and total RNA was isolated with TRIzol reagent (Takara) following the manufacturer’s protocol. RNA concentration and integrity were checked spectrophotometrically. Target cDNA was synthesized and expression was quantified by SYBR Green real-time PCR (Takara Perfect Real Time) on a Bio-Rad MyiQ system, with β-actin as the internal reference. Fold-change was calculated by the 2-ΔΔCt method, normalizing each sample to the mean Ct of the control group set to 1. Genes and primers are listed as follows: The sequences of the SNRPA1 primers were 5’-ACTTGATCAGGCTCTGCCCT-3’ (forward) and 5’-GAGGGTCCAGATCACCCAGT-3’ (reverse). The sequences of the TIGIT primers were 5’-GGTCCTAGAAAGCTCAGTGGC-3’ (forward) and 5’-TTCTAGTCAACGCGACCACC-3’ (reverse). The sequences of the human β-actin primers were 5′- TGGCACCCAGCACAATGAA-3′ (forward) and 5′-CTAAGTCATAGTCCGCCTAGAAGCA-3′ (reverse).

### Drug prediction and molecular docking

We used the Drug Signatures Database (DSigDB, http://dsigdb.tanlab.org/DSigDBv1.0/) to predict these core genes with P<0.05 as the threshold and performed molecular docking for the significant drugs. The protein structure of genes was retrieved from the Protein Data Bank (PDB)(https://www.rcsb.org/), while the 3D or 2D structure of the drug was acquired from PubChem (https://pubchem.ncbi.nlm.nih.gov/). Finally, CB-Dock2 (https://cadd.labshare.cn/cb-dock2/index.php) was utilized to carry out molecular docking. A ΔG value of < -5 kcal/mol is considered indicative of potential binding activity (Liu et al., 2015).

### Machine learning algorithms

A range of ML models, including XGBoost, LightGBM, Random Forest (RF), Adaboost, and SVM were developed and refined by applying a 5-fold cross-validation methodology (Yan et al., 2021; Chen and Guestrin, 2016). XGBoost was an advanced supervised learning technique that enhances predictive accuracy by combining multiple weak models while managing model complexity. LightGBM was a high-performance boosting framework developed by Microsoft that refines traditional gradient boosting methods. RF was a collective learning method that constructs a classification model by integrating multiple decision trees. AdaBoost was an iterative method that strengthens weak classifiers by iteratively adjusting the weights of training instances, giving more emphasis to misclassified instances. Support Vector Machine (SVM) was a classification technique that minimizes structured risk to enhance generalization capabilities. (The final hyperparameter settings for each model are as follows:XGBoost Model was configured with a maximum depth (max_depth) of 6, 300 decision trees (n_estimators), a learning rate of 0.01, and a subsample ratio of 0.8. All other parameters remained at their default values.

LightGBM Model was parameterized as follows: 31 leaf nodes (num_leaves), no restriction on maximum depth (max_depth = -1), a learning rate of 0.05, 200 boosting iterations (n_estimators), and an early stopping mechanism triggered after 20 rounds without improvement (early_stopping_rounds). Other parameters were set to their default values.

RF Model, the number of decision trees (n_estimators) was set to 1000, with no restriction on tree depth (max_depth = NULL). The number of features considered for each split (mtry) was set to the square root of the total number of features (√p), and the minimum node size (min_node_size) was 1. All remaining parameters used default configurations.

AdaBoost Model was configured with 500 weak learners (n_estimators), a learning rate of 0.05, and the exponential loss function (loss = “exponential”). All other parameters were kept at their default values.

SVM Model utilized a radial basis function (RBF) kernel, with a regularization parameter (cost) of 1 and a kernel coefficient (gamma) set to 1/ncol (where ncol represents the number of feature dimensions). Class weights were set to “balanced” to handle potential class imbalance issues. All other parameters remained at their default settings.).

The performance of these models was assessed using various metrics, including accuracy, sensitivity, specificity, positive predictive value, negative predictive value, F1 score, AUC, calibration curve, Decision curve analysis (DCA). The training set was utilized for selecting the optimal model, and the testing set was employed for model test. To enhance the transparency and interpretability of the model, the Shapley Additive Interpretation (SHAP) method was employed to interpret the predicted results and elucidate the impact of each feature on the predictions, thereby offering a practical reference for clinicians. Finally, nomogram was constructed using the biomarkers to predict the risk for obstructive ventilatory dysfunction combined with NTM infection.

### Statistical analysis

R language (version: 4.3.0) and IBM SPSS Statistics software (version: 20.0.0) were used for statistical analysis and visualization. Data are presented as the mean ± standard deviation (SD), with all measurement data conforming to a normal distribution. Two-group comparisons of continuous variables were performed via t tests. The Kruskal-Wallis test and Wilcoxon test were used as nonparametric tests. All continuous variables were first screened for normality with the Shapiro–Wilk test (n < 50) or the Kolmogorov–Smirnov test (n ≥ 50). Consequently, the predictive model used z-scores derived from rank-based inverse normal transformation of non-normal gene-expression values, ensuring that all input features satisfied the distributional assumptions of the subsequent machine-learning algorithms. P<0.05 indicated a statistically significant difference.

## Results

### Identification of DEGs

**Figure 1:**
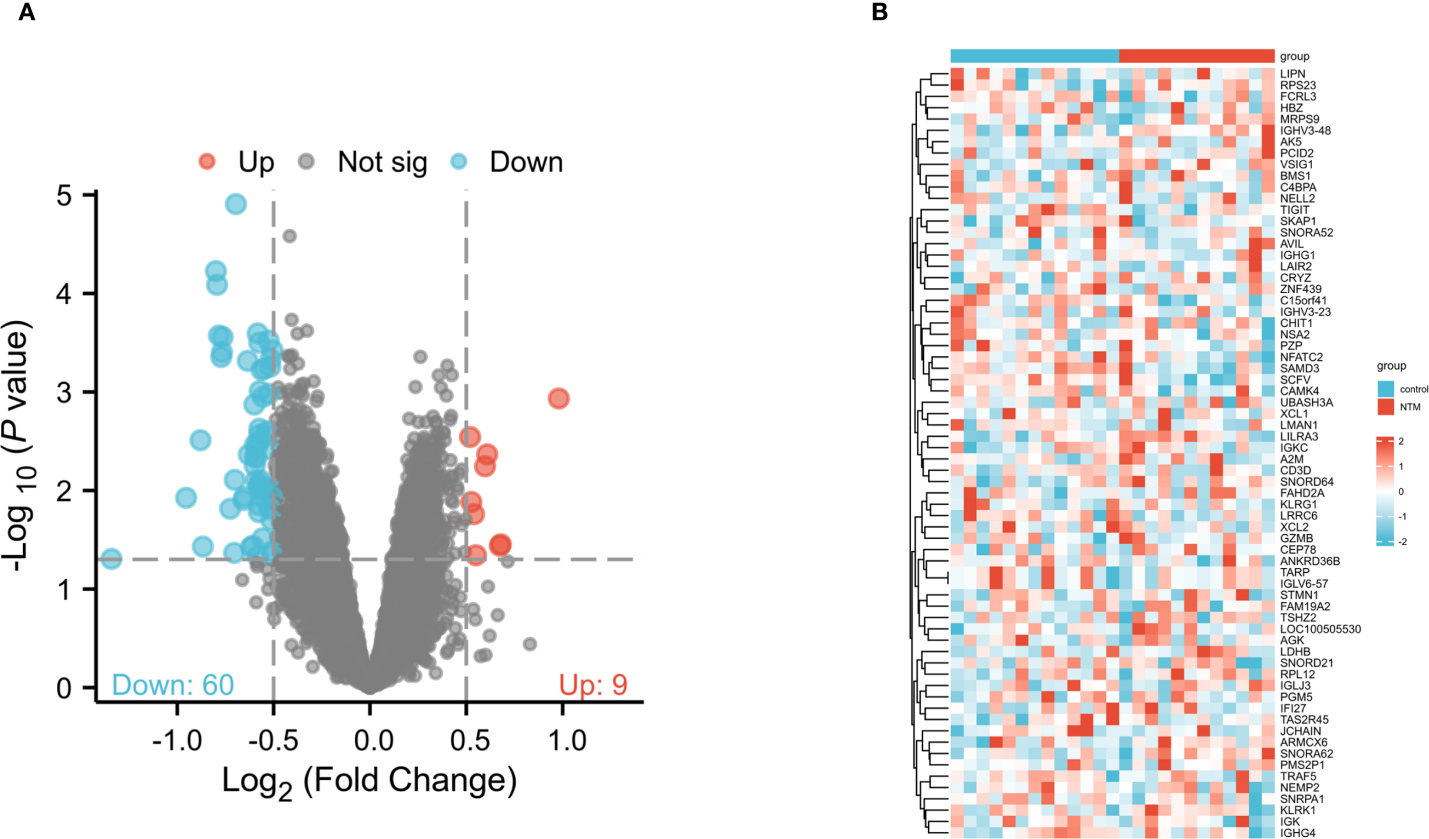
Identification of DEGs between NTM and control group in the GSE97298 microarray set with screening criteria of |logFC|≥ 0.5 and pvalue<0.05. **(A)** Volcano plot of DEGs. The upregulated genes are marked in red; the downregulated genes are marked in blue. **(B)** Heatmap showing DEGs between NTM and control group.

**Figure 2:**
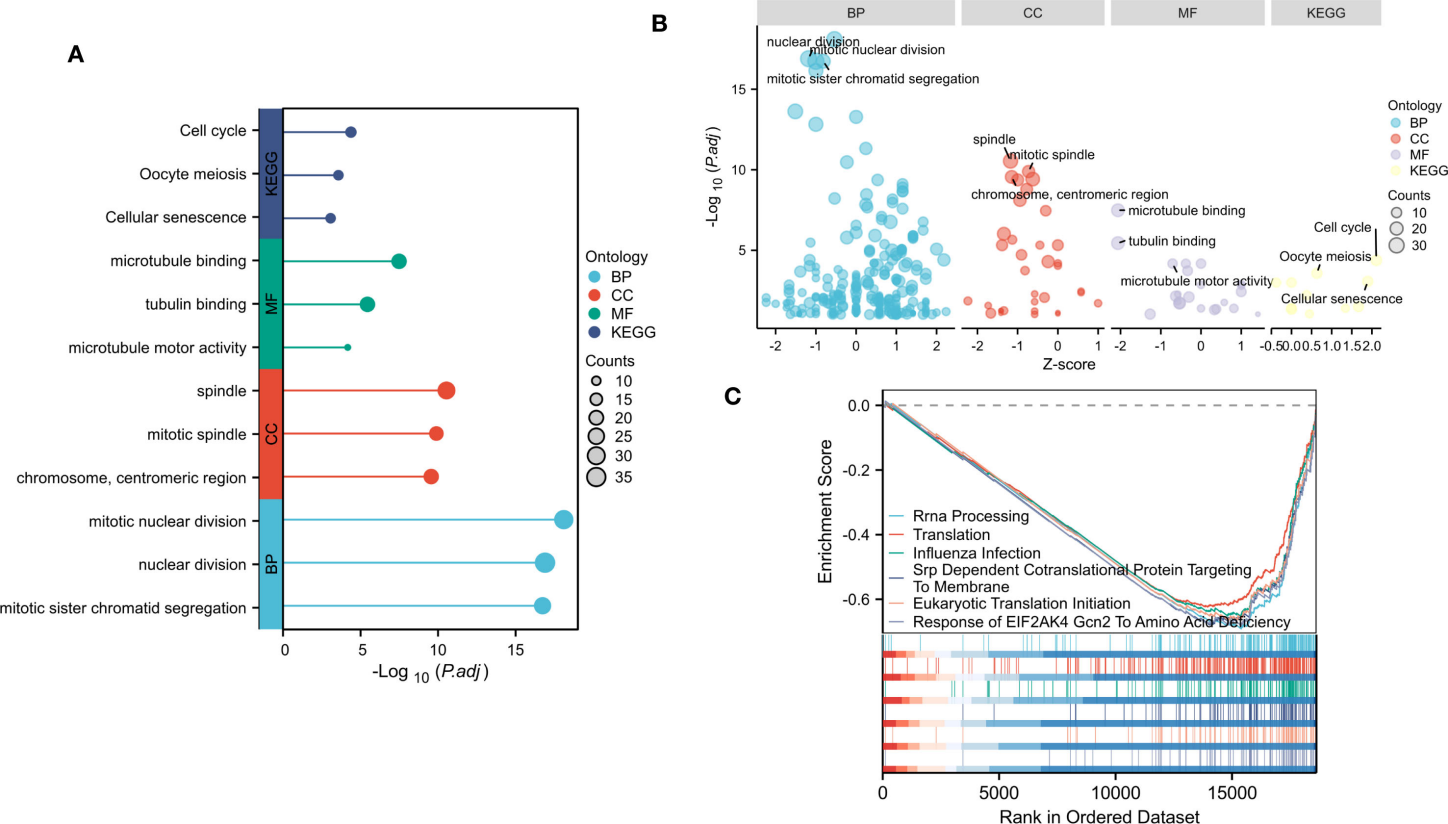
Gene enrichment analysis of DEGs. **(A)** Top 3 significantly enriched GO terms associated with CC, BP, MF and KEGG pathways. **(B)** Bubble plot of enriched GO and KEGG pathways. **(C)**Top six negative enrichment terms of GSEA results.

To explore differentially expressed genes (DEGs) in control and NTM group, we obtained the raw gene expression data from GSE97298 in the GEO database. Following data pre-processing and normalization, a total of 69 DEGs were identified, comprising 60 downregulated and 9 upregulated genes (**Figure 1A**). The DEGs ranked by logFC are displayed in a heatmap (**Figure 2B**). Comprehensive details of all DEGs are provided in **Supplementary Table S1**.

### Gene enrichment analysis of DEGs

To determine the main biological functions of these DEGs, we performed GO and KEGG enrichment analyses on these identified DEGs with or without logFC. GO analysis was performed on the biological processes (BP), cellular components (CC), and molecular functions (MF) of the DEGs. The results revealed that these DEGs were involved mainly in the chromosome, centromeric region, spindle and mitotic spindle (CC). These DEGs are involved mainly in BP related to mitotic nuclear division, nuclear division and mitotic sister chromatid segregation. In addition, these DEGs were enriched in important MF, such as tubulin binding, microtubule binding, and microtubule motor activity. According to the KEGG enrichment analysis, the DEGs play a significant role in the cell cycle, Oocyte meiosis and cellular senescence (**Figures 2A, B**).

To determine the influence of these genes on the phenotype, We performed GSEA analysis on the NTM and control group (**Figure 2C**). The results indicated that top six were all negatively enriched terms and were associated with rRNA processing, translation, influenza infection, srp dependent co translational protein targeting to membrane, eukaryotic translation initiation and response to EIF2AK4 Gcn2 to amino acid deficiency.

### PPI network analysis of DEGs

**Figure 3:**
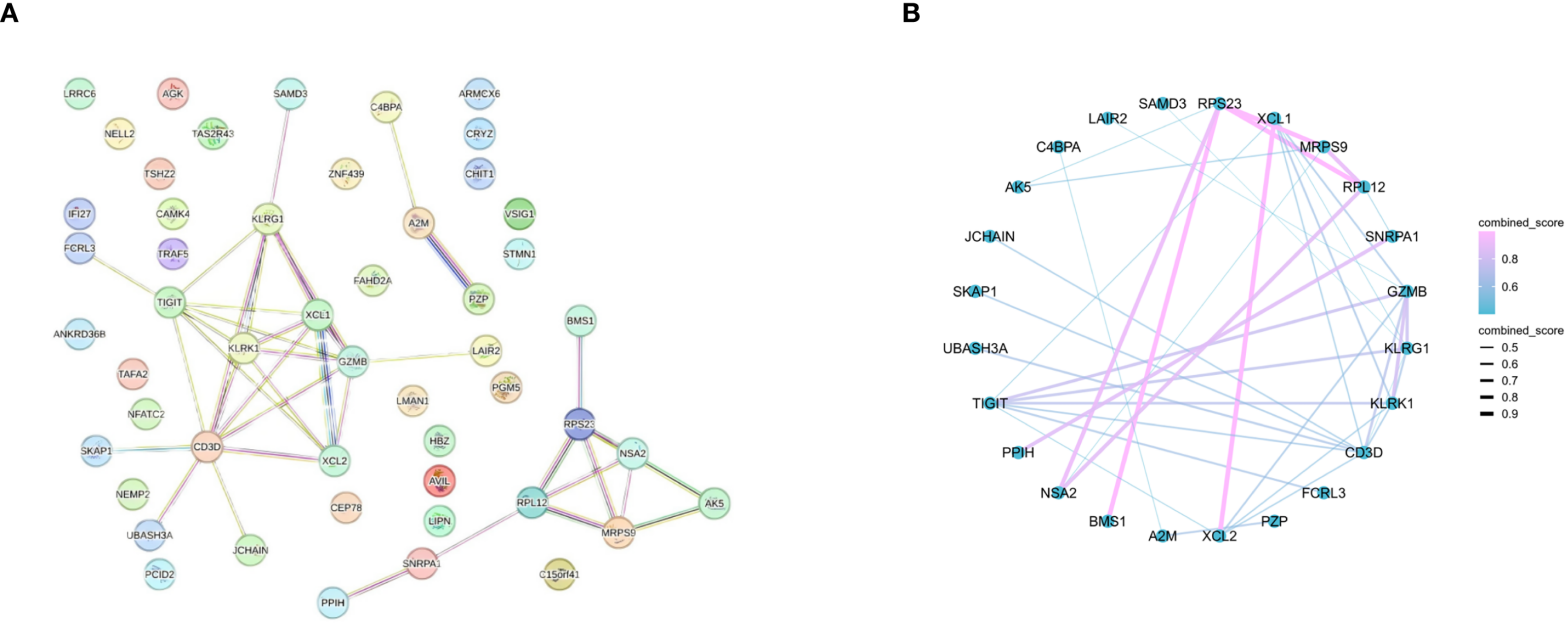
PPIs between DEGs. **(A)** PPI network (69 DEGs filtered into the PPI network that contained 24 nodes and 39 edges). **(B)** PPI network with combined score.

To screen for core genes, we entered the DEGs into the STRING database for PPI network construction and ultimately identified 24 nodes and 39 interactions (**Figures 3A, B**).

### Identification of the core genes and PCR validation

**Figure 4:**
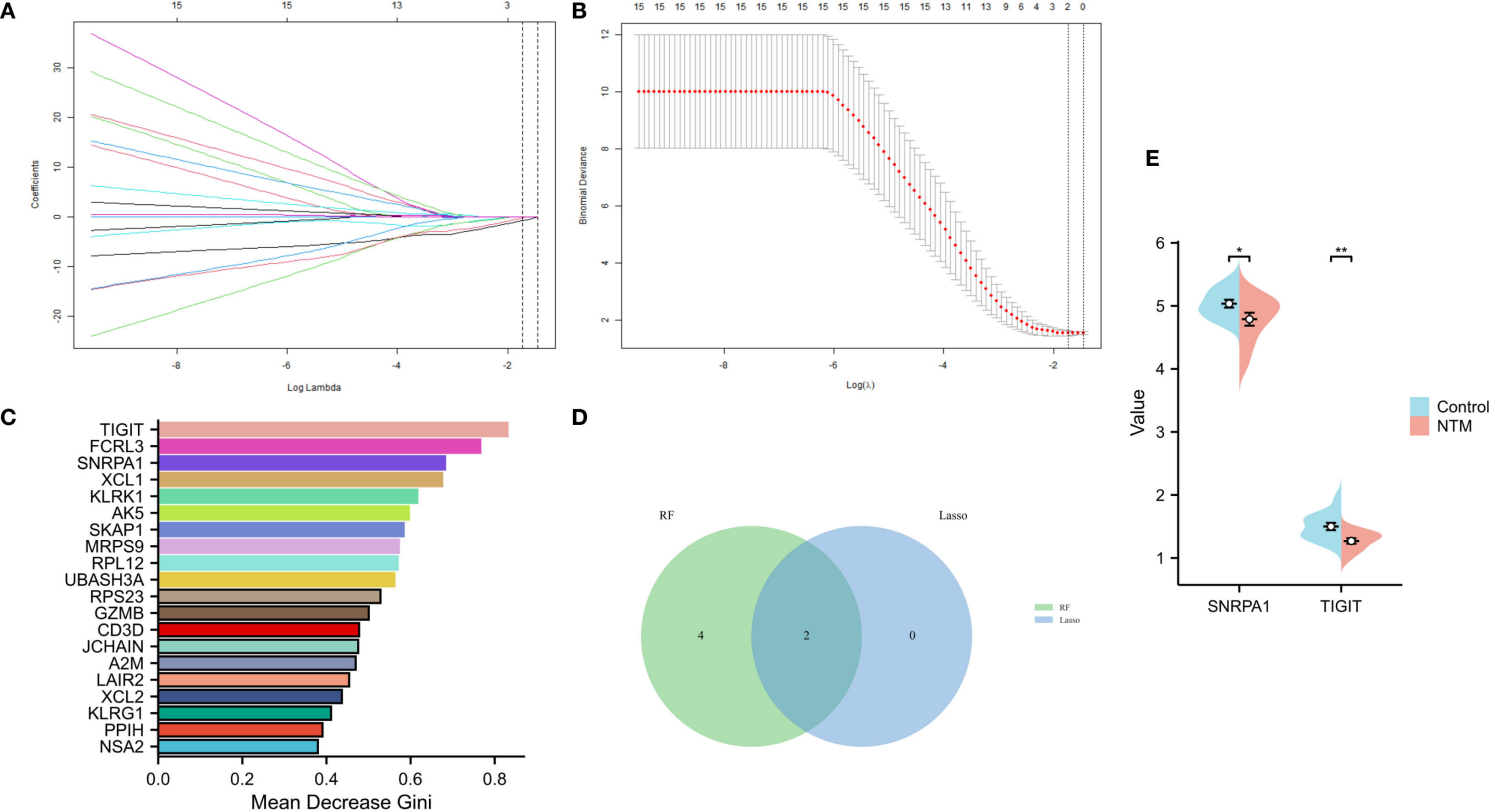
Screening of core genes via two machine learning methods. **(A)** LASSO regression model screening variable trajectories; **(B)** Factor screening based on the LASSO regression model, with the left dashed line indicating the best lambda value for the evaluation metrics (lambda. min) and the right dashed line indicating the lambda value for the model where the evaluation metrics are in the range of the best value by one standard error (lambda.1se). **(C)** 6 core genes with an importance greater than 0.6 were screened via the random forest algorithm. **(D)** Venn diagram displaying the overlap of genes screened by the two machine learning algorithms. **(E)** The core gene in GSE97298 showed significant differences (*p < 0.05, **p < 0.01).

On the basis of the 24 candidate core genes screened by the STRING database, LASSO regression and the random forest method were used to screen core genes related to disease progression. As shown in **Figures 4A, B**, LASSO regression identified two core genes (TIGIT and SNRPA1) through a 10-fold cross-validation procedure. Another 6 core genes (score>0.6 TIGIT, SNRPA1, FCRL3, XCL1, KLRK1, AK5) were screened using the random forest method (**Figure 4C**). Ultimately, two core genes (TIGIT and SNRPA1)were confirmed as shared by both the LASSO regression and random forest algorithms after intersecting the results (**Figure 4D**). In addition, we validated the analytical results in GSE97298 dataset. The findings revealed that TIGIT and SNRPA1 expression were significantly down-regulated in NTM groups (**Figure 4E**). We also performed PCR validation of the core genes, and the results were consistent with the expression profiles observed in the dataset (**Supplementary Figure S2**).

### Prediction of candidate drugs

**Figure 5:**
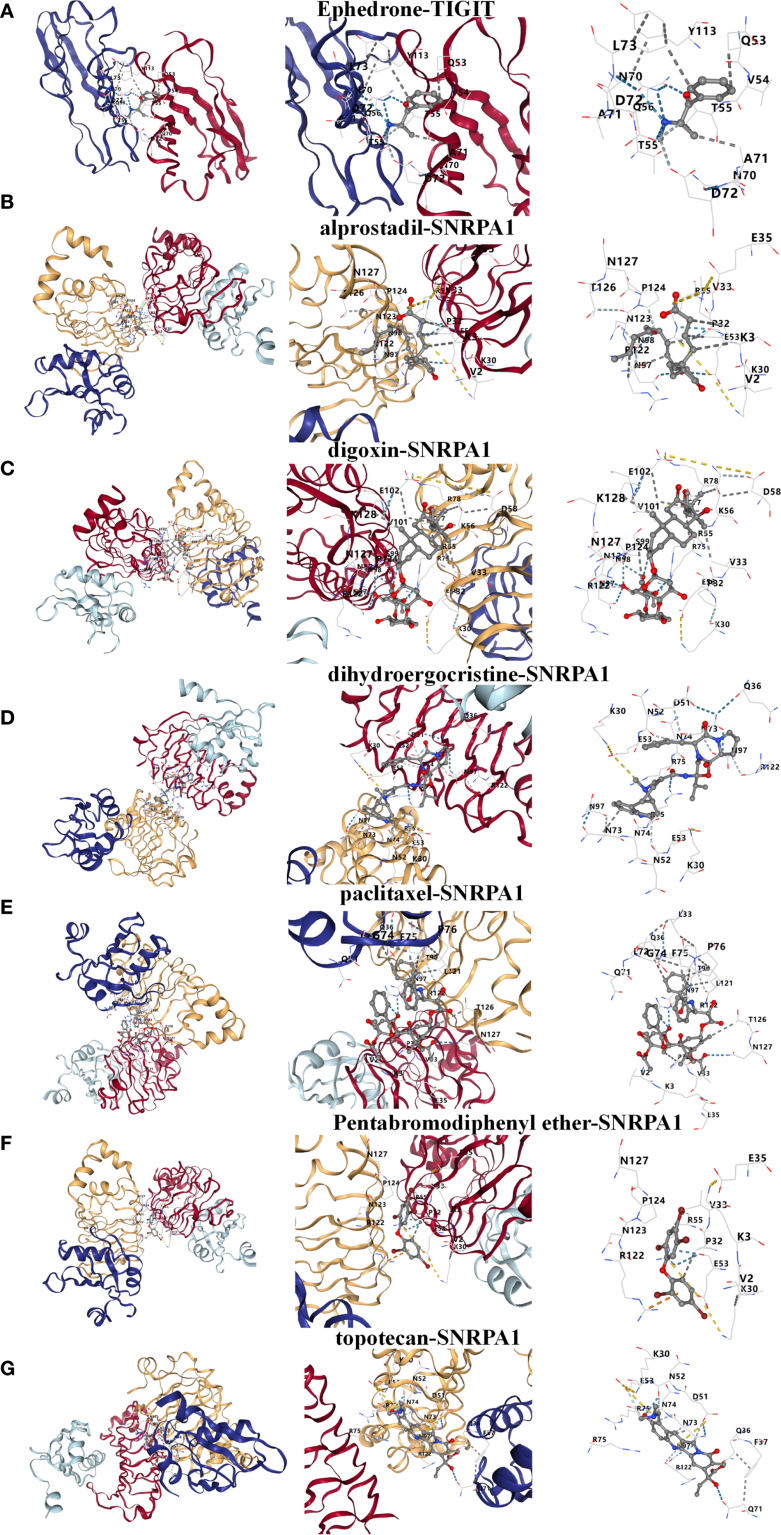
Prediction and molecular docking validation of the candidate drugs based on TIGIT or SNRPA1. **(A-G)** Ephedrone, alprostadil, digoxin, dihydroergocristine, paclitaxel, Pentabromodiphenyl ether and topotecan.

Based on the above studies, We then consulted the DSigDB database to pinpoint drugs with potential efficacy in targeting these core genes. Based on the P value<0.05, a total of 8 drugs including Ephedrone, 2-Bromo-3-hydroxy-4-methoxybenzaldehyde, dihydroergocristine, topotecan, Pentabromodiphenyl ether, digoxin, paclitaxel and alprostadil were selected (**Table 1**). we then used the core genes for molecular docking validation to study the binding with eight drugs (**Figures 5A-G**). Molecular docking verification confirmed that except for 2-Bromo-3-hydroxy-4-methoxybenzaldehyde, the other seven drugs had low binding energy to TIGIT or SNRPA1 (**Table 2**).

**Table 1:** The potential drugs of hub genes were identified using DSigDB.

Term	Pvalue	Combined score	Genes
Ephedrone	0.00379	3006.932	TIGIT
2-Bromo-3-hydroxy-4-methoxybenzaldehyde	0.01275	156.464	SNRPA1
dihydroergocristine	0.01910	410.413	SNRPA1
topotecan	0.02484	293.072	SNRPA1
Pentabromodiphenyl ether	0.04107	151.017	SNRPA1
digoxin	0.04439	135.915	SNRPA1
paclitaxel	0.04869	119.819	SNRPA1
alprostadil	0.04937	117.552	SNRPA1

**Table 2:** Binding energy of 8 candidate drugs.

Drugs	Ephedrone	2-Bromo-3-hydroxy-4-methoxybenzaldehyde	dihydroergocristine	topotecan	Pentabromodiphenyl ether	digoxin	paclitaxel	alprostadil
Binding energy (kcal/mol)	-5.1	-4.9	-8.4	-7.9	-5.8	-9.0	-8.3	-5.3

### Evaluation and analysis of immune cell infiltration using ssGSEA

**Figure 6:**
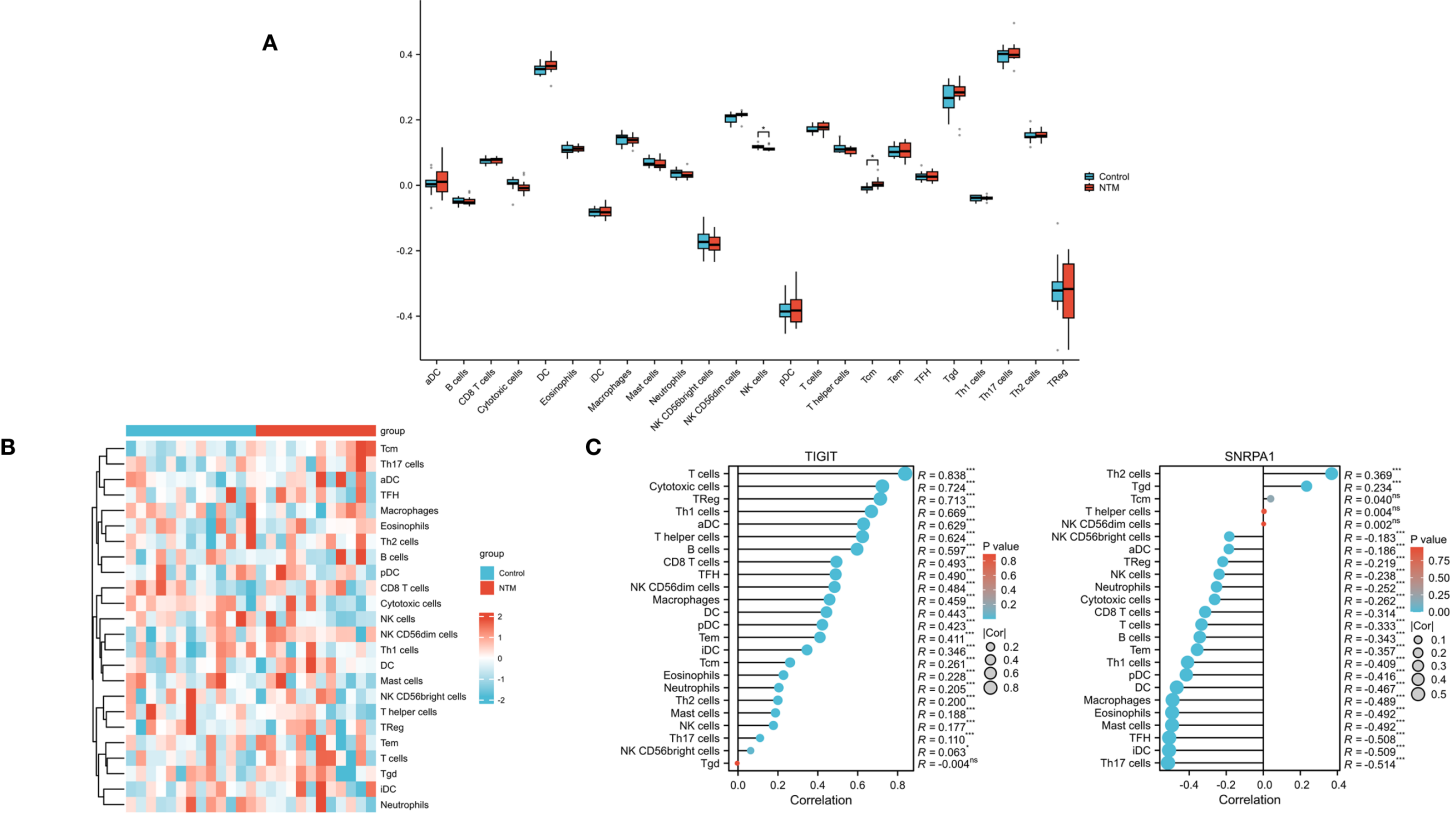
Immune infiltration analysis **(A)** Heatmap showed the composition of 24 kinds of immune cells in each sample. **(B)** Comparison regarding the 24 kinds of immune cells between NTM and control group. **(C)** The correlations between the biomarkers (TIGIT and SNRPA1) and infiltrating immune cells.(*p < 0.05).

To further investigate immune infiltration differences between individuals with NTM and control group, we performed ssGSEA. **Figure 6A** illustrates the distribution of the immune cell types in the dataset. Notably, NTM samples exhibited significantly different infiltration levels of several immune cell types, especially in NK cells and Tcm, compared to controls. This suggests that these immune cell types play a central role in NTM development (**Figure 6B**). Furthermore, the expression of TIGIT is positively correlated with almost all immune cell types (**Figure 6C**). And the expression of SNRPA1 is positively correlated with Th2, Tgd and Tcm cell types.

### Construction of predictive models using machine learning

**Figure 7:**
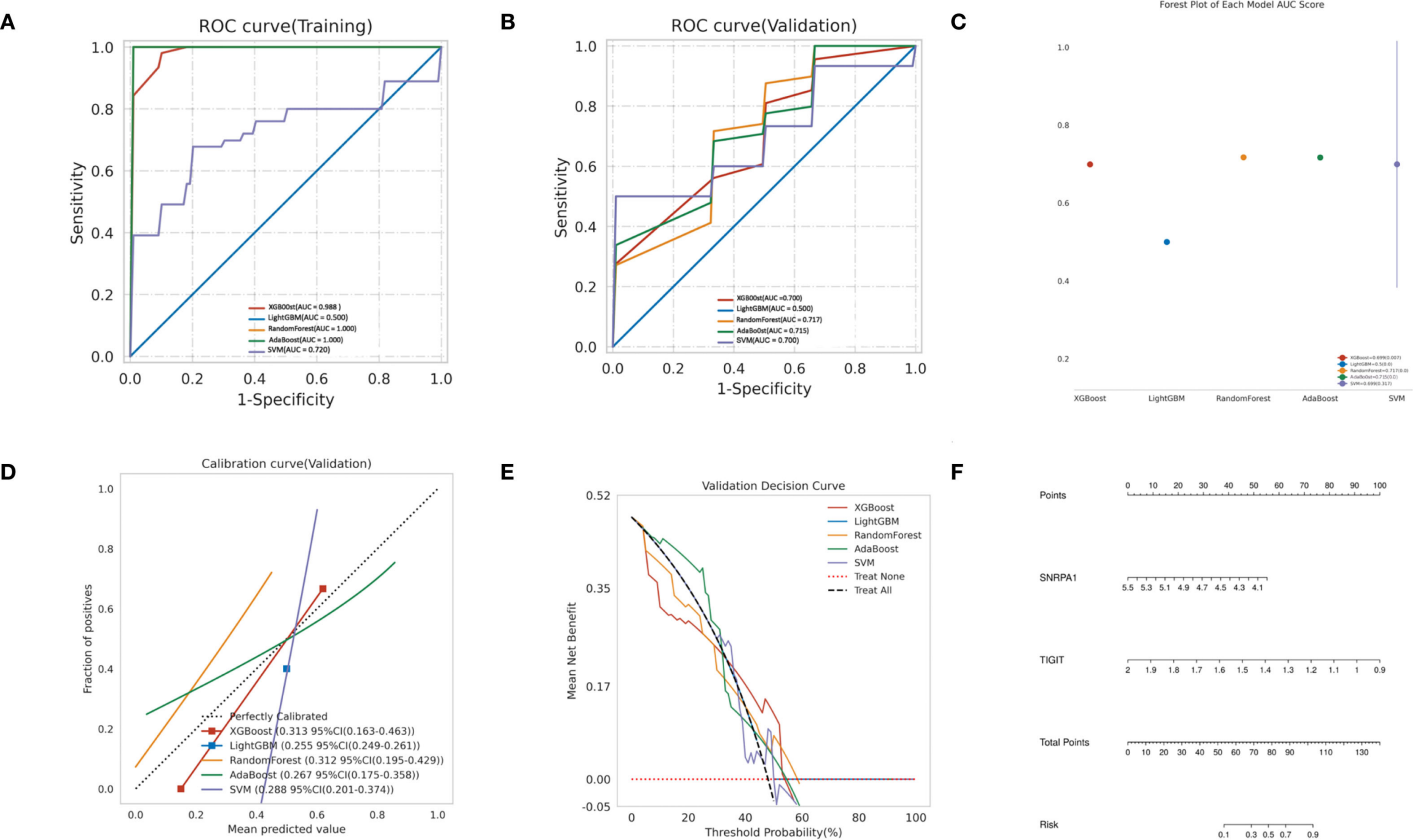
Construction of predictive models using machine learning **(A)** ROC curve comparison of training set in multiple machine algorithms; **(B)** ROC curve comparison of validation set in multiple machine algorithms; **(C)** Forest plot of AUC values in validation set; **(D)** Calibration curve comparison of validation set in multiple machine algorithms; **(E)** Decision curve analysis (DCA) comparison of validation set in multiple machine algorithms. **(F)** Nomogram to predicting obstructive ventilatory dysfunction combined with NTM infection.

In order to establish a predictive model for predicting obstructive ventilatory dysfunction combined with NTM infection based on machine learning algorithms, we use the two core genes (TIGIT and SNRPA1) identified through screening as independent factors we (split the data into a training set and a test set at a 7:3 ratio). The algorithms employed include XGBoost, LightGBM, RF, Adaboost, and SVM. To mitigate overfitting and select the optimal model, 5-fold cross validation was performed using the training set, yielding average values for accuracy, sensitivity, specificity, positive predictive value, negative predictive value, F1 score, and AUC for the five machine learning models (**Supplementary Table S2**). The ROC curves for these models in the training set (**Figure 7A**) and validation set (**Figure 7B**) are illustrated. The forest plot comparing the AUC scores of these ML models in validation set is presented in **Figure 7C**. The comparison of multiple machine learning evaluation indicators (calibration curve, Decision curve) in the validation set is shown in **Figures 7D, E**. The results indicate that, for the training set (AUC = 1.000), the RF model demonstrated superior predictive performance and for the validation set (AUC (95% CI) of 0.717 (0.180-1.000)). We chose the RF model as the optimal model. Finally, a nomogram was generated to predicting obstructive ventilatory dysfunction combined with NTM infection (**Figure 7F**).

### Model validation and SHAP-based model interpretability analysis

**Figure 8:**
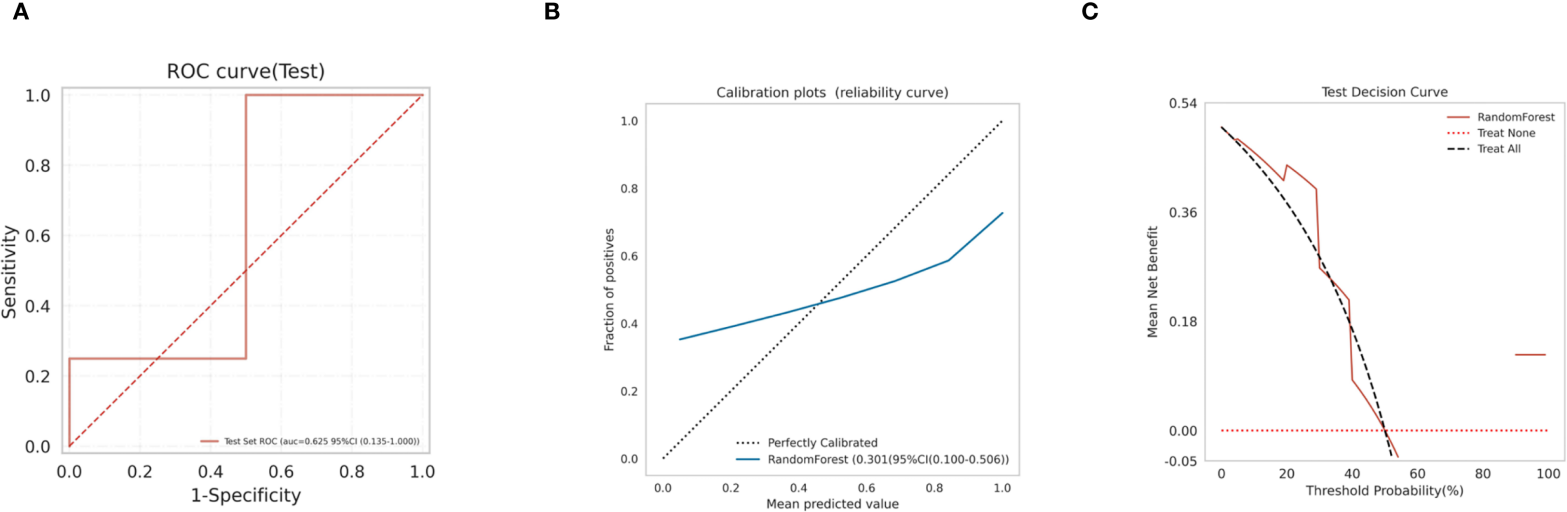
Model validation **(A)** RF model test set ROC curve; **(B)** RF model test set calibration curve; **(C)** RF model test set decision curve analysis (DCA).

Subsequently, We use the test set to validate the model. the ROC curves (**Figure 8A**) for RF model in the test set was analyzed indicting the AUC (95% CI) of 0.625(0.135-1.000), and the calibration curve of the model was also analyzed, demonstrating alignment with the diagonal line, indicative of moderate performance in the test set (**Figure 8B**), with a Brier Score of 0.301. The decision curve analysis (DCA) curve for the RF model also exhibited favorable net clinical benefit, confirming its performance in the test set (**Figure 8C**).

Utilizing SHAP summary plots (**Supplementary Figure S3A**), we computed the contribution of each feature to the model output to identify the most relevant predictive factors. SHAP summary plots visually represents this ranking, with each point denoting a sample and the color gradient from blue to red indicating the magnitude of the sample eigenvalues. The vertical axis displays the importance ranking of features, along with the correlation and distribution of each eigenvalue with the SHAP value. The SHAP importance plot (**Supplementary Figure S3B**) further elucidates the impact of individual features on the model. To enhance comprehension of the model’s decision making process at the individual level, we conducted a detailed interpretability analysis on two representative samples, as illustrated in **Supplementary Figure S3C**. Our findings associate positive predicting outcomes with these factors: lower expression of TIGIT and SNRPA1 genes.

## Discussion

Previous studies have revealed that pNTM infection disease typically occurs in individuals with preexisting obstructive ventilatory dysfunction. Recently, transcriptomic investigations have revealed comprehensive gene expression profiles associated with NTM infection (Cowman et al., 2018; Prieto et al., 2022). These findings suggest that there are significant differences in genomic composition and function among patients with NTM. Although these studies identified several genes associated with NTM, more research is needed given the complexity of this disease. In addition, no relevant studies have been found about molecular signatures linked to obstructive ventilatory dysfunction combined with NTM infection. Thus, we successfully identified the core genes associated with obstructive ventilatory dysfunction combined with NTM infection in the present study by exploring GEO databases. And we constructed the predictive model based on the core genes using machine learning. Consequently, the results obtained are anticipated to offer a more comprehensive understanding of this disease.

We first performed an exhaustive bioinformatics analysis of gene expression profiles to identify key DEGs associated with obstructive ventilatory dysfunction combined with NTM infection. A total of 69 DEGs were identified, comprising 60 downregulated and 9 upregulated genes. Subsequent enrichment analyses aimed to reveal the functional significance of all DEGs, which were found to be involved in processes such as tubulin binding, microtubule binding, and microtubule motor activity. All of the above pathways are related to microtubule dynamics, suggesting that—against the backdrop of NTM infection—various modes and regulatory mechanisms could further perturb microtubule motility and stability, thereby disrupting the balance between contraction and relaxation in airway smooth muscle. Additionally, KEGG analysis highlighted the correlations of all DEGs with the cell cycle, Oocyte meiosis and cellular senescence signaling pathways. GSEA analysis highlighted enriched terms were associated with rRNA processing, translation. Several studies have shown that the occurrence and progression of NTM infection are related to immune cells infiltration, inflammation cell migration and accelerated immune cellular senescence, These findings emphasize the close correlation between the identified DEGs and obstructive ventilatory dysfunction combined with NTM infection (Burzyńska et al., 2025; Chancharoenthana et al., 2025). Subsequently, 24 node genes were identified from the DEGs via the PPI network. The key genes were screened via LASSO and random forest machine learning algorithms. Finally, TIGIT and SNRPA1were selected as the core genes.

There are also 45 DEGs without PPIs in our study play distinct roles in OVD+NTM pathogenesis via multiple pathways, despite lacking direct protein binding. Mucosal barrier-related genes (e.g., LMAN1, AVIL) increase NTM susceptibility: LMAN1 downregulation impairs mucus glycoprotein transport, reducing respiratory mucus secretion, while AVIL reduction disrupts lung epithelial integrity, facilitating NTM penetration. Metabolic regulators like LDHB promote NTM survival—its upregulation enhances lactate accumulation in OVD’s hypoxic environment, inhibiting macrophage bactericidal function. Humoral immunity genes (IGHG1, IGKC, IGLV6-57) indicate activated B-cell responses, yet their efficacy is compromised by OVD-induced mucosal secretion defects, failing to restrict NTM spread. CHIT1 and IFI27 mediate specific anti-NTM defenses: CHIT1 upregulation degrades NTM cell wall chitin, while IFI27 overexpression reflects type I interferon pathway activation for early immune surveillance. Small nucleolar RNAs (SNORA52, SNORD21) exacerbate ribosomal dysfunction by impairing rRNA modification, aligning with GSEA-observed rRNA processing suppression. Collectively, these non-PPI DEGs form an indirect regulatory network linking barrier disruption, metabolic dysregulation, and suboptimal immunity, complementing the core PPI network to clarify OVD+NTM pathophysiology comprehensively.

Previous studies have demonstrated a close relationship between immune cells and NTM infections. Therefore, we analyzed the immune cell infiltration using ssGSEA in obstructive ventilatory dysfunction combined with NTM infection patients. These results exhibited significantly different infiltration levels of several immune cell types, especially in NK cells and Central Memory T cells (Tcm), compared to controls. The two core genes also showed good correlation with a variety of immune cells. This suggests that these immune cell types play a central role in NTM development. Studies have shown that NK cells can identify lipid components in the mycobacterial cell wall through their natural cytotoxicity and produce IFN and IL-22. These cytokines can activate the fusion of phagosomes with lysosomes, thereby inhibiting the intracellular growth of mycobacteria (Cruz-Aguilar et al., 2021; Gramegna et al., 2022). Tcm cells can rapidly differentiate into effector T cells and produce large amounts of cytokines, thereby enhancing the host’s immune defense (Abe et al., 2020; Ratnatunga et al., 2022). These findings emphasize the close correlation between the immune cells and obstructive ventilatory dysfunction combined with NTM infection. However, ssGSEA cannot distinguish between the limitations of cell-subset abundance and activation state. Follow-up *in vitro* functional assays will be needed to validate these cellular mechanisms.

This study validated TIGIT (a T/NK cell checkpoint molecule) and SNRPA1 (a U1 snRNP splicing factor) as core diagnostic biomarkers for obstructive ventilatory dysfunction (OVD) combined with non-tuberculous mycobacteria (NTM) infection. The mechanistic relevance of these markers to OVD+NTM pathophysiology lies in their roles in immune regulation and RNA processing, respectively.​For TIGIT, its significant downregulation in OVD+NTM patients’ peripheral CD4+ T cells reflects a disrupted immune compensation loop. In isolated OVD, mild TIGIT upregulation typically restricts excessive pulmonary inflammation driven by chronic hypoxia and oxidative stress (Zhang et al., 2022). However, NTM infection reverses this trend: NTM’s cell wall components (e.g., lipoarabinomannan) trigger TIGIT reduction to enhance anti-pathogen immunity, yet this compensation is ineffective due to NTM’s ability to inhibit macrophage phagosome maturation and antigen presentation via virulence factors like ESAT-6 (Naser et al., 2020).​ SNRPA1 changes in OVD+NTM lung tissues may drive pathogenesis by dual mechanisms: promoting pro-inflammatory cytokine splicing (e.g., IL-1β, TNF-α) and mediating lung epithelial stress repair. However, its functional efficacy is constrained by GSEA-observed downregulation of rRNA processing/translation, which impairs spliceosome assembly—since SNRPA1’s splicing activity depends on co-expression of U1 snRNP components synthesized via ribosomal pathways (Pacheco et al., 2020). Key limitations include the absence of *in vitro* validation (e.g., siRNA-mediated SNRPA1 knockdown in NTM-infected cells) and an OVD-only control group, which are critical for confirming biomarker specificity and functional roles in future work.

Currently, the treatment of NTM infections is still primarily based on antibiotics. However, the side effects caused by combination therapy and the emergence of drug resistance make it difficult for many patients to adhere to their medication regimens. Therefore, the need for differential medication requires further exploration. Based on the above studies, we then consulted the DSigDB database to pinpoint drugs with potential efficacy in targeting these core genes. And selecting 7 candidate drugs using molecular docking validation finally.

We further constructed the predictive model based on the core genes using five machine learning models, including XGBoost, LightGBM, RF, AdaBoost and SVM. Taking all factors into consideration, we finally chose the RF model as the optimal model. The thorough evaluation of the model’s performance using metrics and visualizations, such as ROC curves, calibration curves, and decision curve analysis, adds credibility to the study’s findings. The application of the Random Forest model as the final classification model for the test set yielded promising results, emphasizing its potential utility in predicting obstructive ventilatory dysfunction combined with NTM infection. However, the random forest model shows significant overfitting, with training AUC = 1.000 dropping to 0.625 on the independent validation set (ΔAUC > 0.3). This limitation is attributable to the overall small sample size. In future studies, we will include a larger cohort to obtain more definitive results. In clinical contexts, the interpretability of ML models is crucial. Our study utilized the SHAP (SHapley Additive exPlanations) technique to provide a comprehensive understanding of the ML model, spanning both overarching trends and detailed individual predictions. This enhanced the model’s visual representation and transparency. Compared to conventional interpretation methods that depend on weights, the SHAP approach has demonstrated greater reliability and efficiency, and it remains effective across various models. Through SHAP value analysis, we obtained a novel viewpoint on the model’s decision-making process, enabling us to pinpoint the unique contribution of each predictor to the model’s outputs. Ultimately, this significantly elevated the model’s interpretability and clarity. In addition, a nomogram was also constructed to facilitate the application of the model.

This study also has several limitations. Firstly, this model was constructed based on a single center retrospective study and only have 25 samples in the set (some clinical information were also missing), which inevitably suffered from confounding bias and model overfitting; Secondly, an independent validation is very important for determining the clinical usefulness of a predictive model; therefore, whether the proposed model is applicable to other centers needs further validation. Third, the candidate drugs identified through our network-pharmacology screening will require *in-vivo* or *in-vitro* validation in future studies. Further studies should involve larger sample sizes, multi center prospective studies, or randomized controlled trials (RCTs) to further validate our findings.

## Conclusions

In conclusion, our research indicates that TIGIT and SNRPA1 were closely associated with obstructive ventilatory dysfunction combined with NTM infection, serving as one of the important driving factors for the development of this disease. In addition, the study successfully leveraged machine learning algorithms, particularly the Random Forest model, to develop a predictive model for obstructive ventilatory dysfunction combined with NTM infection. The robust performance of the model suggests its potential clinical utility in guiding treatment decisions and improving outcomes for patients.

## Data Availability

The original contributions presented in the study are included in the article/**Supplementary Material**. Further inquiries can be directed to the corresponding authors.

## Supplementary material

Supplementary Figure 1 The flow diagram of study design.

Supplementary Figure 2 Quantification of mRNA expression by real-time RT-PCR. mRNA levels are shown as arbitrary units normalized to β-actin expression. n = 3, *p<0.05 versus control.

Supplementary Figure 3 SHAP-based model interpretability analysis **(A)** SHAP dendrogram of features of this model; **(B)** Importance ranking plot of features; **(C)** Interpretability analysis of 2 independent samples.

## Glossary

NTM: nontuberculous mycobacteria
pNTM: pulmonary nontuberculous mycobacteria
OVD: obstructive ventilatory dysfunction
GEO: Gene Expression Omnibus
DEGs: differentially expressed genes
GO: Gene Ontology
KEGG: Kyoto Encyclopedia of Genes and Genomes
GSEA: Gene Set Enrichment Analysis
PPI: protein–protein interaction
LASSO: Least Absolute Shrinkage and Selection Operator
RF: Random Forest
ssGSEA: single-sample Gene Set Enrichment Analysis
PCR: polymerase chain reaction
RT-PCR: reverse transcription polymerase chain reaction
DSigDB: Drug Signatures Database
PDB: Protein Data Bank
ML: machine learning
XGBoost: eXtreme Gradient Boosting
LightGBM: Light Gradient Boosting Machine
Adaboost: Adaptive Boosting
SVM: Support Vector Machine
SHAP: Shapley Additive exPlanations
ROC: receiver operating characteristic
AUC: area under the curve
DCA: decision curve analysis
