# Supplementary material for: TIGIT and SNRPA1 as novel diagnostic and predictive biomarkers in obstructive ventilatory dysfunction combined with pulmonary nontuberculous mycobacterial infection patients
Source: Front Cell Infect Microbiol. 2025 Oct 1;15:1621129. doi: 10.3389/fcimb.2025.1621129 (PMC12521183; doi:10.3389/fcimb.2025.1621129)
Supplement: Supplementary file 2 [file Table2.docx]

Training set

| Methods | AUC(95%CI) | accuracy(95%CI) | sensitivity(95%CI) | specificity(95%CI) | positive predictive value(95%CI) | negative predictive value(95%CI) | F1 score(95%CI) | Methods | AUC(95%CI) |
| --- | --- | --- | --- | --- | --- | --- | --- | --- | --- |
| XGBoost | 0.988 (0.960-1.000) | 0.616(0.564-0.668) | 0.94(0.920-0.960) | 0.96(0.912-1.008) | 0.925(0.858-0.993) | 0.925(0.858-0.993) | 0.964(0.920-1.007) | 0.939(0.920-0.958) | 0.88(0.842-0.919) |
| LightGBM | 0.500 (0.132-0.632) | 1.48(1.456-1.504) | 0.52(0.496-0.544) | 0.0(0.000-0.000) | 1.0(1.000-1.000) | nan(NaN-NaN) | 0.52(0.496-0.544) | nan(NaN-NaN) | 0.0(0.000-0.000) |
| RandomForest | 1.000 (NaN-NaN) | 0.64(0.556-0.724) | 1.0(1.000-1.000) | 1.0(1.000-1.000) | 1.0(1.000-1.000) | 1.0(1.000-1.000) | 1.0(1.000-1.000) | 1.0(1.000-1.000) | 1.0(1.000-1.000) |
| AdaBoost | 1.000 (NaN-NaN) | 0.668(0.655-0.682) | 1.0(1.000-1.000) | 1.0(1.000-1.000) | 1.0(1.000-1.000) | 1.0(1.000-1.000) | 1.0(1.000-1.000) | 1.0(1.000-1.000) | 1.0(1.000-1.000) |
| SVM | 0.720 (0.578-0.841) | 0.728(0.333-1.124) | 0.78(0.650-0.910) | 0.698(0.348-1.047) | 0.844(0.745-0.942) | nan(NaN-NaN) | 0.806(0.658-0.953) | nan(NaN-NaN) | 0.54(0.245-0.835) |

Validation set

| Methods | AUC(95%CI) | accuracy(95%CI) | sensitivity(95%CI) | specificity(95%CI) | positive predictive value(95%CI) | negative predictive value(95%CI) | F1 score(95%CI) | Methods | AUC(95%CI) |
| --- | --- | --- | --- | --- | --- | --- | --- | --- | --- |
| XGBoost | 0.700 (0.145-1.000) | 0.616(0.564-0.668) | 0.56(0.413-0.707) | 0.533(0.090-0.976) | 0.633(0.270-0.997) | nan(NaN-NaN) | nan(NaN-NaN) | nan(NaN-NaN) | 0.156(-0.077-0.390) |
| LightGBM | 0.500 (NaN-NaN) | 1.48(1.456-1.504) | 0.52(0.424-0.616) | 0.0(0.000-0.000) | 1.0(1.000-1.000) | nan(NaN-NaN) | 0.52(0.424-0.616) | nan(NaN-NaN) | 0.0(0.000-0.000) |
| RandomForest | 0.717 (0.180-1.000) | 0.64(0.556-0.724) | 0.6(0.476-0.724) | 0.533(0.141-0.925) | 0.733(0.352-1.114) | nan(NaN-NaN) | nan(NaN-NaN) | nan(NaN-NaN) | 0.237(0.014-0.461) |
| AdaBoost | 0.715 (0.178-0.998) | 0.668(0.655-0.682) | 0.56(0.413-0.707) | 0.4(0.019-0.781) | 0.733(0.352-1.114) | nan(NaN-NaN) | nan(NaN-NaN) | nan(NaN-NaN) | 0.107(-0.213-0.428) |
| SVM | 0.700 (0.145-1.000) | 0.728(0.333-1.124) | 0.52(0.363-0.677) | 0.3(-0.092-0.692) | 0.8(0.408-1.192) | nan(NaN-NaN) | nan(NaN-NaN) | nan(NaN-NaN) | 0.109(-0.105-0.323) |
