# Supplementary figures and images for: TIGIT and SNRPA1 as novel diagnostic and predictive biomarkers in obstructive ventilatory dysfunction combined with pulmonary nontuberculous mycobacterial infection patients
Source: Front Cell Infect Microbiol. 2025 Oct 1;15:1621129. doi: 10.3389/fcimb.2025.1621129 (PMC12521183; doi:10.3389/fcimb.2025.1621129)

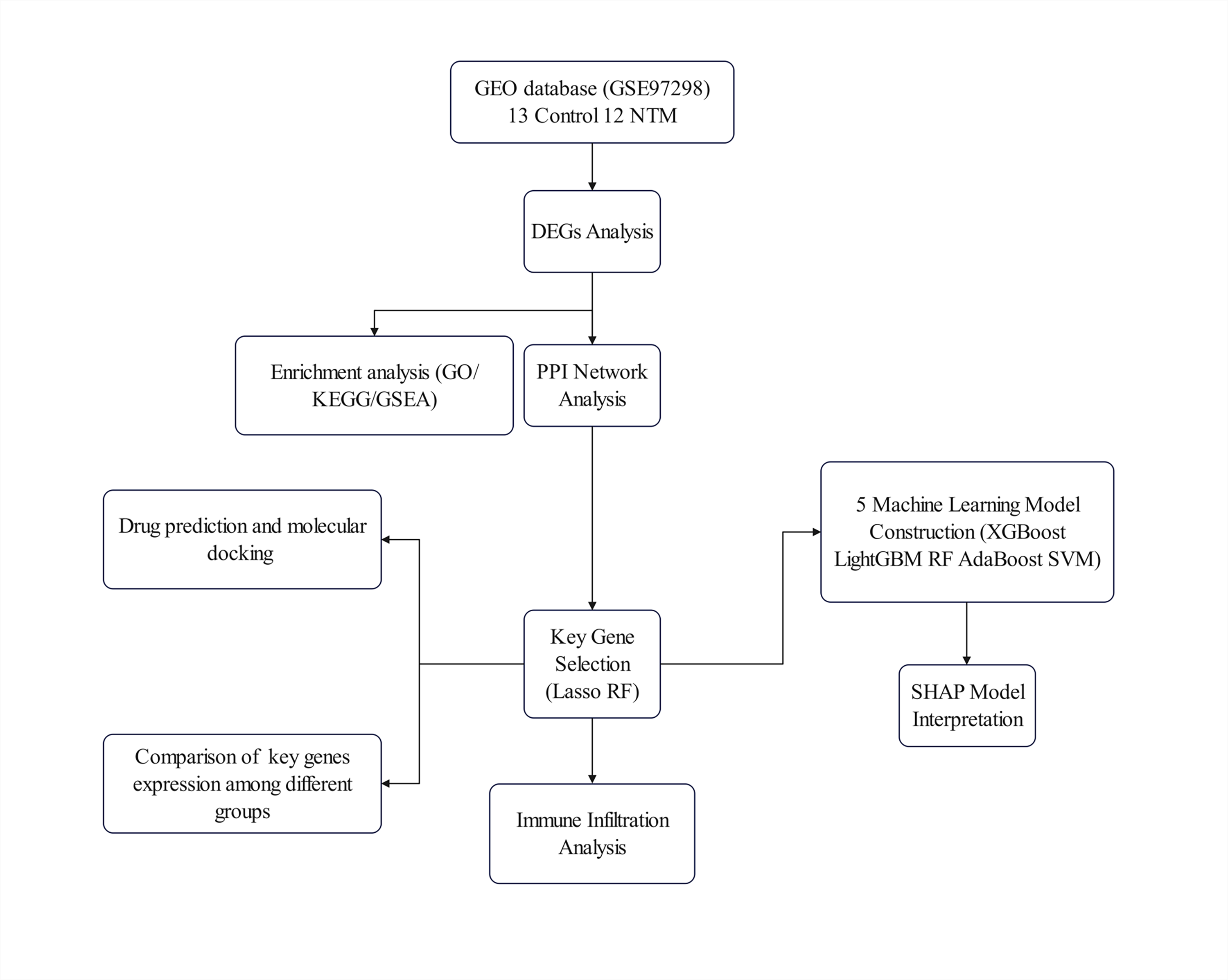

Supplement: Supplementary Figure 1 — The flow diagram of study design. [file Image1.tif]

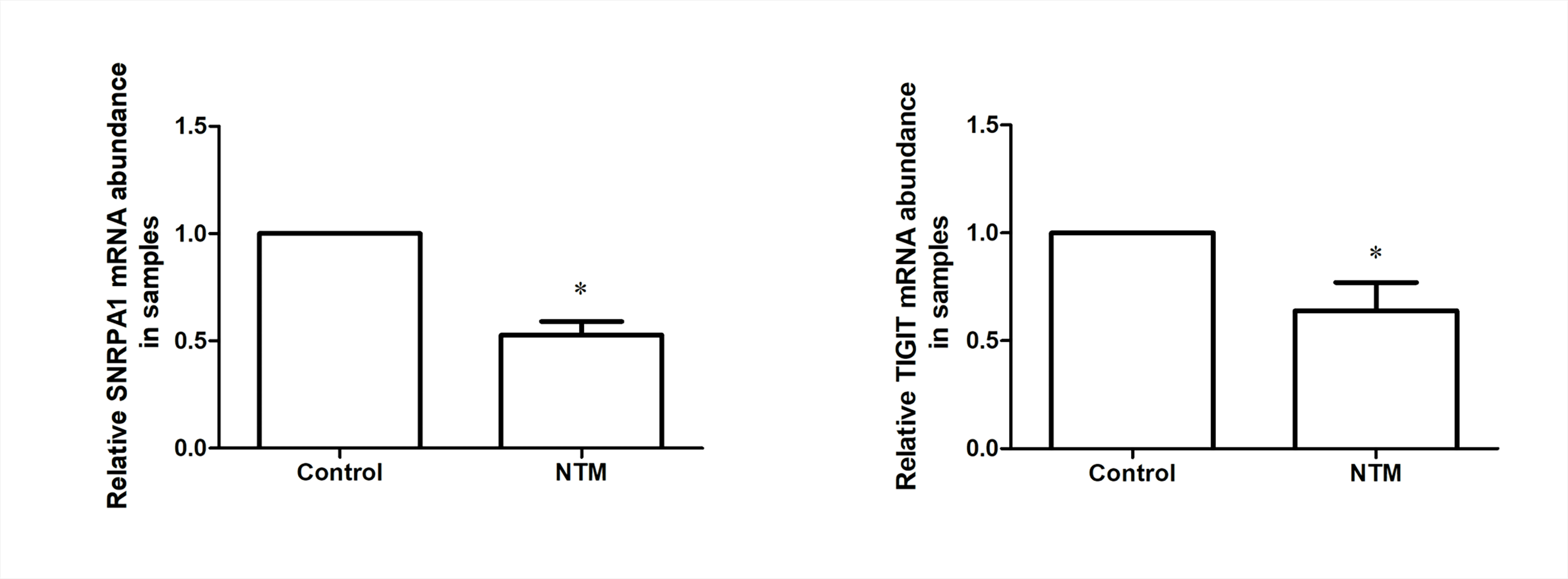

Supplement: Supplementary Figure 2 — Quantification of mRNA expression by real-time RT-PCR. mRNA levels are shown as arbitrary units normalized to β-actin expression. n = 3, *p<0.05 versus control. [file Image2.tif]

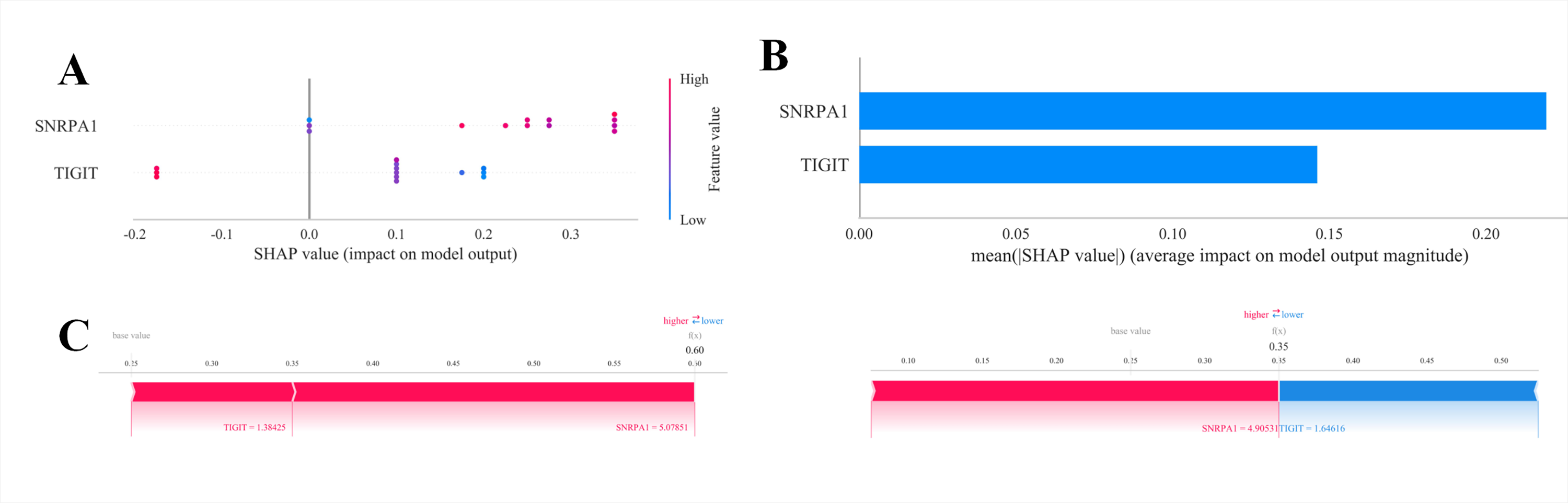

Supplement: Supplementary Figure 3 — SHAP-based model interpretability analysis (A) SHAP dendrogram of features of this model; (B) Importance ranking plot of features; (C) Interpretability analysis of 2 independent samples. [file Image3.tif]
